# The Detection of the Pipe Crack Utilizing the Operational Modal Strain Identified from Fiber Bragg Grating

**DOI:** 10.3390/s19112556

**Published:** 2019-06-04

**Authors:** Zechao Wang, Mingyao Liu, Yongzhi Qu, Qin Wei, Zude Zhou, Yuegang Tan, Liu Hong, Han Song

**Affiliations:** 1 School of Mechanical and Electronic Engineering, Wuhan University of Technology, Wuhan 430070, China; whutwzc@whut.edu.cn (Z.W.); quwong@whut.edu.cn (Y.Q.); zudezhou@whut.edu.cn (Z.Z.); ygtan@whut.edu.cn (Y.T.); hongliu@whut.edu.cn (L.H.); songhan@whut.edu.cn (H.S.); 2 Hubei Key Laboratory of Digital Manufacturing, Wuhan University of Technology, Wuhan 430070, China; 3 School of Information Engineering, Wuhan University of Technology, Wuhan 430070, China

**Keywords:** structural health monitoring, crack detection, operation modal strain, Fiber Bragg Grating, enhanced damage indicator

## Abstract

The small and light-weight pipeline is widely used in hydraulic system for aerospace engineering. The crack is one of the most common failures in the pipelines so that its incipient detection can further avoid the catastrophic damage of the piping system. The electrical and piezoelectric sensors are conventionally used for the structural health monitoring (SHM), while these are not suitable for the cascaded pipelines in harsh environment because the added mass will change the modal characteristics of the cascaded pipelines. The Fiber Bragg Grating (FBG) sensor with light-weight, multiplexed, and anti-electromagnetic interference properties, are employed to obtain the modal strain transmissibility with a novel diagram of the operational modal analysis (OMA). Based on the OMA an enhanced damage indicator is proposed to detect the crack. After going through analytical modeling, finite element modeling (FEM) and its corresponding experiments, it is concluded that the presented method is effective and accurate to detect and locate the crack.

## 1. Introduction

The small and light-weight pipeline is widely used in hydraulic system for aerospace engineering; however, its cracking causes the catastrophic damage of the whole system, which is one of the most common failures in the pipelines. Current research aims to detect the incipient crack for the cascaded pipeline, and generally are based on the dynamic characteristics, i.e., natural frequencies, deflection mode shapes, modal damping, FRF and Curvature Mode Shapes (CMS) et.al. Extensive literature reviews about the state of the art in methods for detecting, localizing and characterizing damage by examining changes in the vibration characteristics are in Refs [1,2,3].

In these methods mentioned above, the natural frequencies are the most attractive as it is relatively easy to measure [4,5,6,7,8]. Compared with it, the appealing feature of mode shapes is that its distributing characteristic allows customers to assess damage with high spatial resolution. Nguyen investigated the interaction mechanism between the horizontal bending and vertical bending vibration due to the crack on the deflection mode shapes and the characteristics of the mode shapes are further employed for the crack localization [9]. To enhance the performance of mode shapes in the smaller crack detection, Solis et al. applied the continuous wavelet transform (CWT) to analyze the mode shape differences [10]. To improve its sensitivity to damages the deflection slope and curvature mode shapes (CMS) have been presented in several literatures [11,12,13,14,15]. Yam et al. have demonstrated that the strain mode shape (SMS) is more sensitive to the crack than the deflection mode shape [16]. Since the slope of the deflection and the CMS are difficult to measure directly, some studies aim to present a modal strain based method to detect the cracks in pipelines for its relatively easier measurement.

The sensing technology is another hot topic in the SHM for the piping system. Yan et al. developed a finite element model using piezoelectric elements to detect the pipeline damage [17]. Guan et al. presented a multiple mode nonlinear guided waves to detect the fatigue crack [18]. Du et al. presented a time reversal technique to detect the corrosion pits on the pipelines with a piezo-ceramic transducer as a time reversal mirror [19]. Zhu et al. proposed a method to locate the impact on underwater pipelines using a determination technique for both arrival-time and group velocity of ultrasonic waves with lead zirconate titanate (PZT) transducers [20]. Zuo et al. presented a modified electromechanical impedance (EMI) technique for crack detection [21]. However, there are some drawbacks in the piezoelectric transducers based methods, i.e., saturation and electromagnetic interference, which make it difficult to apply to the harsh environments. The wiring is another problem for the electronic sensors. The Fiber Bragg Grating (FBG) sensor with light-weight, multiplexed, and anti-electromagnetic interference properties has been employed for the SHM of the pipelines. Wang et al. utilized the FBG to measure the dynamic strain of the hydraulic piping system and compared the dynamic properties of the FBG and electronic accelerators. The experimental results showed that the effects of the added mass of the accelerators changed the modal properties of the pipelines [22]. Li et al. analyzed the stain properties of the pretension high-strength concrete (PHC) pipe piles under hydraulic jacking based on the FBG sensing technology [23]. Ren et al. designed an FBG based hoop-strain sensor to monitor the pipeline [24]. Then, they applied the FBG based hoop strain senor to detect the pipeline corrosion [25]. Recently, Huang et al. conducted the experimental strain modal analysis (ESMA) for the pipelines based on the FBG sensing technology [26]. Due to the many advantages of the FBG sensor, the FBG sensing technology will be employed in this work.

However, the OMA for the small and light-weight pipeline has not been widely studied. In our previous work, the FBG based OMA has conducted to detect the clamp looseness in the cascaded pipelines and an improved diagram of OMA is presented [27]. The operational modal strain based method has been used to the damage detection in beam and plate structures [28,29,30]. To the best knowledge of the authors, there is no work about the application of the operational modal strain to detect the crack in the small and light-weight pipeline. The main contributions of the paper can be concluded as follows:

(1) An enhanced damage indicator is presented by considering the differences of the natural frequencies and modal strain simultaneously. When compared with the damage indicator presented in [30], the damage indictor presented in this work is weighted by the change ratio of the natural frequencies, which can make the small damages discernible.
(2) The modal strain transmissibility is employed to be equivalent to modal strain in order to exclude the influence of the unknown excitation. Although the transmissibility based method has been presented to detect and locate the damages in the existing literatures [31,32,33], the location of the excitation and the frequency band used to calculate the transmissibility will affect the accuracy of the detection [34]. However, the modal transmissibility used in this work is independent on the excitation locations. The corresponding numerical and experimental results are conducted to validate the proposed method to detect and locate the crack.

The remainder of the paper is formed as follows: The Section 2 gives the introduction of the operational modal parameter identification techniques; the modal strain based crack detection and location method is given in Section 3; Section 4 shows the results and discussions and the conclusions are given in Section 5.

## 2. Operational Modal Parameter Identification Techniques

Performing a modal test on an operating structure is very challenging because the excitation applied on the structure is hard to obtain in practice. In order to overcome these problems, a technique named Natural Excitation Technique (NExT) is introduced in the 1990s. It was a prototype of the well-known OMA for the structure. By utilizing this technique, the inputs do not need to be measured and the modal parameters are estimated only considering the outputs, i.e., accelerations, displacements and strains. Since the main purpose of the paper is to use the modal strain to detect the pipe cracks without measuring the inputs, i.e., forces, the OMA technique has to be used. The simplest method to identify the modal parameters of a structure under the ambient excitation is the peak-picking method and the natural frequencies are identified as the peaks of the power spectrum. If the frequencies are not well separated, the wrong results will be obtained by using this method since it identifies the operational deflection shapes (ODS) instead of the mode shapes. The other drawback is that the selection of natural frequencies is subjective if the power spectrum is not well discernable [35]. A more advanced non-parametric method, i.e., frequency domain decomposition (FDD) has been presented by Brincker et al. [36], which is based on a singular value decomposition (SVD) of the auto and cross power spectral among the outputs. The stochastic subspace identification method is another well-known method [37]. Several parametric methods have been presented for the OMA. A frequency-domain maximum likelihood method has presented by Hermans et al. [38]. The poly-reference complex frequency method is originally presented by Guillaume et al. for the experimental modal analysis [39]. Then they applied the method to the OMA [40]. However, the method needs to fit all the outputs to obtain the mode shapes. Another new technique for the OMA is based on the fact that the transmissibility will converges to the ratio of the mode shapes when the frequency goes to the natural frequencies [41,42]. In order to shorten the calculation cost in Polymax method, an improved method by combing the idea of the Polymax and Transmissibility based method is presented in our previous work [27]. The advantages of the method only a single output is used to obtain the physical poles of the system. Since our main aim is obtaining the natural frequency accurately, the employed method can shorten the time to conduct the modal analysis.

For a continuous system, the strain frequency response function matrix can be expresses as follows:

(1) $${\left[H^{\varepsilon }\right]}_{N_{O\;,}N_{i}}=\overset{M}{\underset{r=1}{\sum }}\frac{1}{\left(k_{r}-\omega^{2}\cdot m_{r}+j\omega \cdot c_{r}\right)}\cdot \left[\begin{array}{ccc} \psi_{1r}^{\varepsilon }\cdot \varphi_{1r} & \psi_{1r}^{\varepsilon }\cdot \varphi_{2r} & \begin{array}{cc} \cdots & \psi_{1r}^{\varepsilon }\cdot \varphi_{Nir} \end{array}\\ \psi_{2r}^{\varepsilon }\cdot \varphi_{1r} & \psi_{2r}^{\varepsilon }\cdot \varphi_{2r} & \begin{array}{cc} \cdots & \psi_{2r}^{\varepsilon }\cdot \varphi_{Nir} \end{array}\\ \begin{array}{c} \vdots \\ \psi_{Nor}^{\varepsilon }\cdot \varphi_{1r} \end{array} & \begin{array}{c} \vdots \\ \psi_{Nor}^{\varepsilon }\cdot \varphi_{2r} \end{array} & \begin{array}{cc} \begin{array}{c} \ddots \\ \cdots \end{array} & \begin{array}{c} \vdots \\ \psi_{Nor}^{\varepsilon }\cdot \varphi_{Nir} \end{array} \end{array} \end{array}\right]$$

where $N_{O}$, $N_{i}$, and M denote the number of outputs, inputs and modes respectively. The element in the *i*th row and *l*th column of the matrix ${\left[H^{\varepsilon }\right]}_{N_{O\;,}N_{i}}$ can be expressed as follows:

(2) $$H_{il}^{\varepsilon }=\overset{M}{\underset{r=1}{\sum }}\frac{\psi_{ir}^{\varepsilon }\cdot \varphi_{ir}}{k_{r}-\omega^{2}\cdot m_{r}+j\omega \cdot c_{r}}\left(i=1,2,\;\cdots ,N_{o};l=1,2,\;\cdots ,N_{i}\right)$$

where $\psi_{ir}^{\varepsilon }$ is the *r*th order strain mode shapes (SMS) of the *i*th output and $\varphi_{ir}$ is the *r*th order displacement mode shapes (DMS) on *l*th output. And $k_{r}$, $m_{r}$, and $c_{r}$ is the *r*th order modal stiffness, modal mass and modal damping ratio. Similar to the decomposition of the displacement frequency response function in references [36], the modal decomposition of the Equation (1) can be obtained as follows:

(3) $$H_{il}^{\varepsilon }\left(j\omega \right)=\overset{M}{\underset{r=1}{\sum }}\left[\frac{\psi_{ir}^{\varepsilon }\cdot L_{lr}^{T}}{j\omega -\lambda_{r}}+\frac{\psi_{ir}^{\varepsilon *}\cdot L_{lr}^{H}}{j\omega -\lambda_{r}^{*}}\right]$$

where $L_{r}^{T}$ is the *r*th modal participation of the *l*th input, the superscript ∗ is the complex conjugate operator, superscript H is the complex conjugate transpose operator, and $\lambda_{r}$ are the *r*th physical poles which can be expressed as follows:

(4) $$\lambda_{r},\;\lambda_{r}^{*}=-\xi_{r}\cdot \omega_{r}\pm j\cdot \sqrt{1-\xi_{r}^{2}}\cdot \omega_{r}$$

where the $\xi_{r}$ is the *r*th order modal damping ratio and $\omega_{r}$ is the *r*th order natural frequency.

In the case of the OMA the output spectra are only information available. The output spectra $\left[S_{yy}^{\varepsilon }\left(\omega \right)\right]$ can be expressed as follows:

(5) $$\left[S_{yy}^{\varepsilon }\left(\omega \right)\right]=\left[H^{\varepsilon }\left(\omega \right)\right]\cdot \left[S_{II}\left(\omega \right)\right]\cdot {\left[H^{\varepsilon }\left(\omega \right)\right]}^{H}$$

where $\left[S_{II}\left(\omega \right)\right]$ is the input spectra. If the input is the white noise, then the $\left[S_{II}\left(\omega \right)\right]$ is constant. Based on the work of Refs. [43,44,45] the output spectra can be converted to partial form as follows:

(6) $$\left[S_{yy}^{\varepsilon }\left(j\omega \right)\right]=\overset{M}{\underset{r=1}{\sum }}\frac{\left\{\psi_{r}^{\varepsilon }\right\}\cdot \langle g_{r}\rangle }{j\omega -\lambda_{r}}+\frac{\left\{{\psi_{r}^{\varepsilon }}^{*}\right\}\cdot \langle g_{r}^{*}\rangle }{j\omega -\lambda_{r}^{*}}+\frac{\left\{g_{r}\right\}\cdot \langle \psi_{r}^{\varepsilon }\rangle }{-j\omega -\lambda_{r}}+\frac{\left\{g_{r}^{*}\right\}\cdot \langle {\psi_{r}^{\varepsilon }}^{*}\rangle }{-j\omega -\lambda_{r}^{*}}$$

where $\langle g_{r}\rangle$ are the operational reference factors. The preprocessing technique needs to be used to transform the operational data. Two well-known methods, i.e., modified Welch’s period-gram and weighted correlogram can be used to estimate the spectrum. The Welch’s method is employed in this paper, which has the following advantages:

(1) It is sufficient to compute the PSPS by using the positive correlations.
(2) The modal decomposition of the PSPS can be obtained as follows:
  (7) $$S_{yy}^{\varepsilon }{\left(j\omega \right)}^{+}=\overset{M}{\underset{r=1}{\sum }}\frac{\left\{\psi_{r}^{\varepsilon }\right\}\cdot \langle g_{r}\rangle }{j\omega -\lambda_{r}}+\frac{\left\{{\psi_{r}^{\varepsilon }}^{*}\right\}\cdot \langle g_{r}^{*}\rangle }{j\omega -\lambda_{r}^{*}}$$
(3) The lower order models can be fitted without affecting the quality.

In our study, the Forsythe complex orthogonal polynomials and the Least squares technique are employed to fit the elements of the Equation (7), which is different from the Polymax since only an elements of the Matrix $\left[S_{yy}^{\varepsilon }{\left(j\omega \right)}^{+}\right]$ is used in our method. Note that the poles calculated from the estimated denominator including the mathematical poles and physical poles, therefore is of great importance to separate the mathematical poles and the physical poles for the users [46]. A stable diagram will be produced, if the variations of the natural frequencies, damping ratios and strain mode shapes with respect to the model order after obtaining the physical poles from the stabilization diagram, the strain response transmissibility (SRT) can be calculated as follows:

(8) $$T_{ij}^{\varepsilon }\left(\omega \right)=\frac{X_{\varepsilon ,i}\left(\omega \right)}{X_{\varepsilon ,j}\left(\omega \right)}$$

where $X_{\varepsilon ,i}\left(\omega \right)$ spectra of the *i*th output and $X_{\varepsilon ,j}\left(\omega \right)$ is the spectra of the *j*th output. When jω goes to the system poles $\lambda_{r}$, then the strain SRT converges to the follows:

(9) $$\underset{j\omega \to \lambda_{r}}{\lim}T_{ij}^{\varepsilon }\left(\omega \right)=\frac{\psi_{ir}^{\varepsilon }\cdot g_{1r}^{T}\cdot F_{1}\left(\omega \right)+\psi_{ir}^{\varepsilon }\cdot g_{2r}^{T}\cdot F_{2}\left(\omega \right)+\cdots +\psi_{ir}^{\varepsilon }\cdot g_{Nir}^{T}\cdot F_{Ni}\left(\omega \right)}{\psi_{jr}^{\varepsilon }\cdot g_{1r}^{T}\cdot F_{1}\left(\omega \right)+\psi_{jr}^{\varepsilon }\cdot g_{2r}^{T}\cdot F_{2}\left(\omega \right)+\cdots +\psi_{jr}^{\varepsilon }\cdot g_{Nir}^{T}\cdot F_{Ni}\left(\omega \right)}\;=\frac{\psi_{ir}^{\varepsilon }\cdot \left[g_{1r}^{T}\cdot F_{1}\left(\omega \right)+g_{2r}^{T}\cdot F_{2}\left(\omega \right)+\cdots +g_{Nir}^{T}\cdot F_{Ni}\left(\omega \right)\right]}{\psi_{jr}^{\varepsilon }\cdot \left[g_{1r}^{T}\cdot F_{1}\left(\omega \right)+g_{2r}^{T}\cdot F_{2}\left(\omega \right)+\cdots +g_{Nir}^{T}\cdot F_{Ni}\left(\omega \right)\right]}=\frac{\psi_{ir}^{\varepsilon }}{\psi_{jr}^{\varepsilon }}$$

From Equation (9), it is clear that the $T_{ij}^{\varepsilon }\left(\omega \right)$ converges to the ratio of the SMS of the *i*th output and *j*th output. And then the following equation can be obtained as follows:

(10) $$\underset{j\omega \to \lambda_{r}}{\lim}\left[T_{1j}^{\varepsilon },T_{2j}^{\varepsilon }\;,\;\cdots ,\;T_{\left(j-1\right)j}^{\varepsilon },1,T_{\left(j+1\right)j}^{\varepsilon },\cdots T_{Noj}^{\varepsilon }\right]=\left[\frac{\psi_{1r}^{\varepsilon }}{\psi_{jr}^{\varepsilon }},\frac{\psi_{2r}^{\varepsilon }}{\psi_{jr}^{\varepsilon }},\cdots ,\frac{\psi_{\left(j-1\right)r}^{\varepsilon }}{\psi_{jr}^{\varepsilon }},1,\frac{\psi_{\left(j+1\right)r}^{\varepsilon }}{\psi_{jr}^{\varepsilon }},\cdots \frac{\psi_{Nor}^{\varepsilon }}{\psi_{jr}^{\varepsilon }}\right]$$

**Figure 1:**
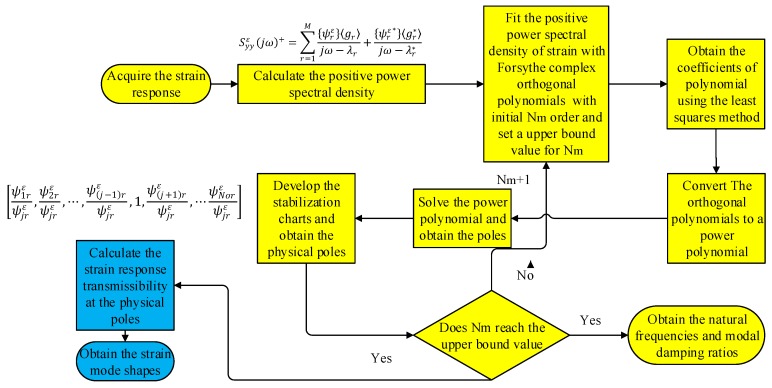
The novel diagram of the presented method for OMA.

The diagram for the presented method of the OMA is shown in Figure 1.

## 3. The Modal Strain Based Crack Detection and Location Method

Yam et al. have demonstrated that the modal strain is more sensitive to the local damages compared with the modal displacement. The damage indicator based on the modal strain presented in the literatures [30] will be discussed in this section, and then an enhanced damage indicator for the detection of the crack is presented.

### 3.1. The Indicator Based on the Difference of the Mode Shapes Presented in [30]

The strain mode shape difference is defined as follows:

(11) $$\psi_{\Delta r}^{\varepsilon }\left(i\right)=\left|\psi_{dr}^{\varepsilon }\left(i\right)-\psi_{hr}^{\varepsilon }\left(i\right)\right|$$

where $\psi_{dr}^{\varepsilon }\left(i\right)$ and $\psi_{hr}^{\varepsilon }\left(i\right)$ denote the rth strain mode shape with respect to the ith element of the structure under damaged and undamaged conditions respectively. Then the difference of the modal strain is normalized as follows:

(12) $$\psi_{N,\Delta r}^{\varepsilon }\left(i\right)=\frac{\psi_{\Delta r}^{\varepsilon }\left(i\right)}{\underset{i}{\underbrace{max\left[\psi_{\Delta r}^{\varepsilon }\left(i\right)\right]}}}$$

Then the averaged normalized modal strain differences for all the M modes can be obtained as follows:

(13) $$Average(\psi_{N,\Delta r}^{\varepsilon }\left(i\right))=\frac{1}{M}\overset{M}{\underset{r=1}{\sum }}\psi_{N,\Delta r}^{\varepsilon }\left(i\right)$$

The damaged elements of the structure can be defined as follows:

(14) $$Damaged\;elemnts=\left\{i:|Average(\psi_{N,\Delta r}^{\varepsilon }\left(i\right))>\sigma \right\}$$

where σ is the threshold which can be determined statistically and the specific method to obtain the threshold σ can be found in Reference [47] And the averaging process decreases the effect of the uncertainty and noise.

### 3.2. The Enhanced Damage Indicator Considering the Variation of the Natural Frequecies

In order to enhance the damage indicator in Equation (13), in what follows, a damage indicator considering the difference of the natural frequencies will be presented.

The natural frequency difference is defined as follows:

(15) $$\omega_{r,\;∆}=\frac{\left|\omega_{r,d}-\omega_{r,h}\right|}{\omega_{r,h}}$$

Then the averaged normalized modal strain difference considering the variation of the natural frequencies is obtained as follows:

(16) $$Average\left(\omega_{r,\;∆}\cdot \psi_{N,\Delta r}^{\varepsilon }\left(i\right)\right)=\frac{1}{M}\overset{M}{\underset{r=1}{\sum }}\omega_{r,\;∆}\cdot \psi_{N,\Delta r}^{\varepsilon }\left(i\right)$$

However, in order to eliminate the influence of the unknown excitation the modal strain transmissibility shown in Equation (10) is employed to calculate the $\psi_{r}^{\varepsilon }$ in present study. This is another contribution of the paper. And the $\psi_{r}^{\varepsilon }$ is normalized with a maximum of 1, the following procedure to calculate the $\psi_{N,\Delta r}^{\varepsilon }\left(i\right)$ is repeated from Equation (11) to Equation (12).

## 4. Results and Discussions

Firstly, in this section, the accuracy of the diagram of OMA will be validated with an analytical model. Secondly, the presented damage indicator will be compared with the indicator shown in Reference [30] by using a finite element model generated in the software ANSYS WORKBENCH. Lastly, the corresponding experiments are conducted to validate the presented method to detect the pipe cracks.

### 4.1. Validate the OMA with an Analytical Model

**Figure 2:**
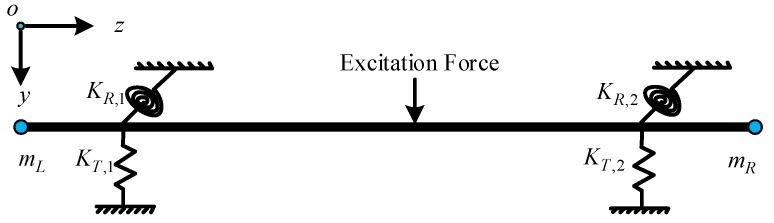
The three-span pipe model.

**Figure 3:**
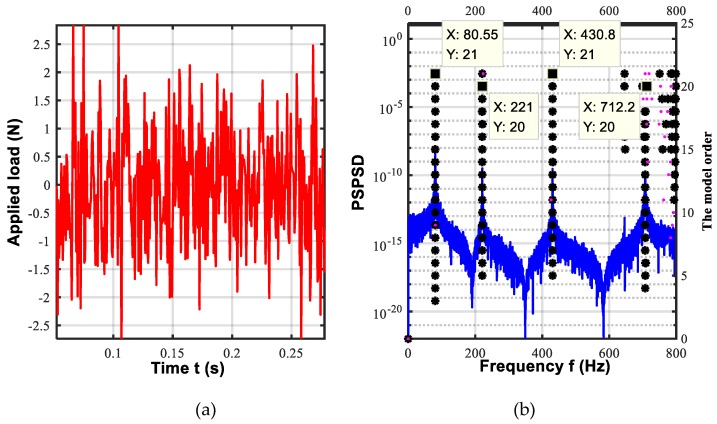
The time series and the stabilization diagram of the output: (**a**) The time series of the excitation; (**b**) The stabilization diagram: black asterisk-frequency variation ratio [0–0.5%], Pinkdot_frequency variation ratio [0.5–1%].

**Figure 4:**
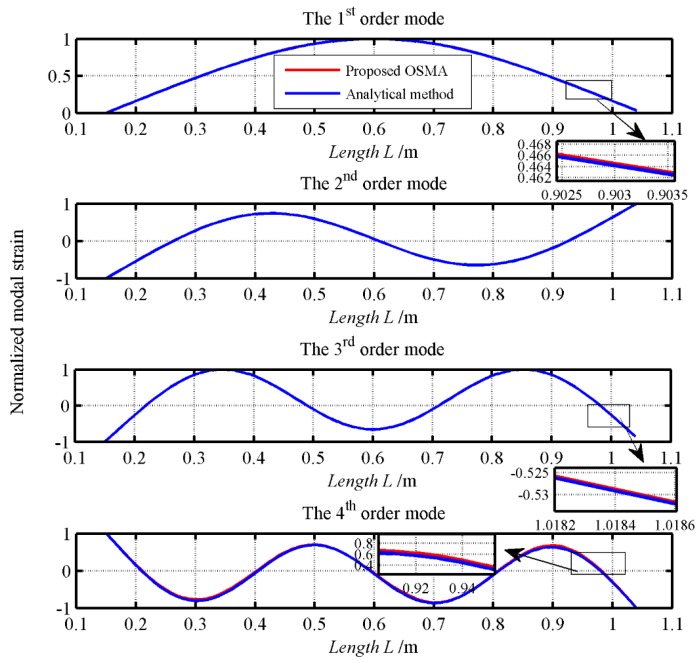
The comparison of the strain mode shapes identified from the OMA and the results calculated from the analytical method.

To validate the presented diagram of OMA, a three-span pipe model shown in Figure 2 is employed. The physical properties of the pipe model are listed in Table 1. The transfer matrix method [48] and pseudo excitation method (PEM) [49] are combined to calculate the PSPD of the pipe model under white noise excitation and the detailed process for the analytical modelling is given in Appendix A. What’s more, from the Appendix A. An Equation (A25) can be obtained analytically. The time series of the excitation shown used in Equation (A28) is shown in Figure 3a. The corresponding stabilization diagram in the operational modal analysis is further shown in Figure 3b. The identified natural frequencies and the damping ratio from the OMA presented in Figure 1 and the ones calculated from the Equation (A25) are listed in Table 2. The comparisons of strain mode shapes estimated from OMA and the results calculated from Appendix A are shown in Figure 4.

In Table 3, it is clear that the maximum relative error of the identified natural frequencies from the OMA and the analytical method is –1.3%. And the damping ratio is close to zero, i.e., the analytical results. In Figure 4, it is obvious that strain mode shapes identified from the OMA and the results from the analytical method are almost consistent. Altogether, the presented OMA is accurate.

### 4.2. The Comparision of the Presented Damage Indicator with the One Presensted in Existing Literature

**Figure 5:**
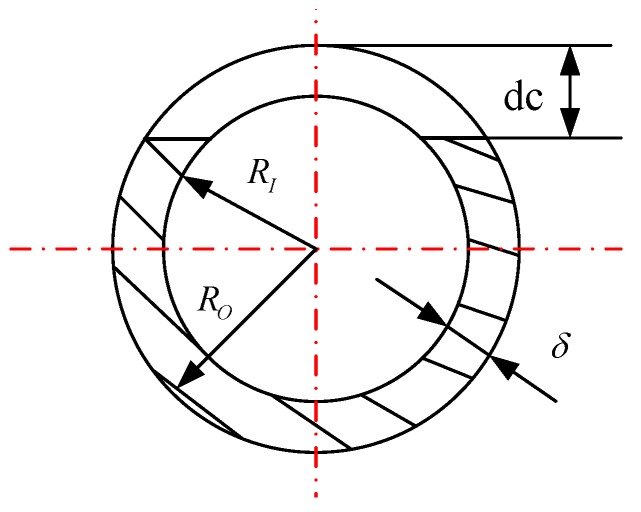
The crack form of the pipeline.

**Figure 6:**
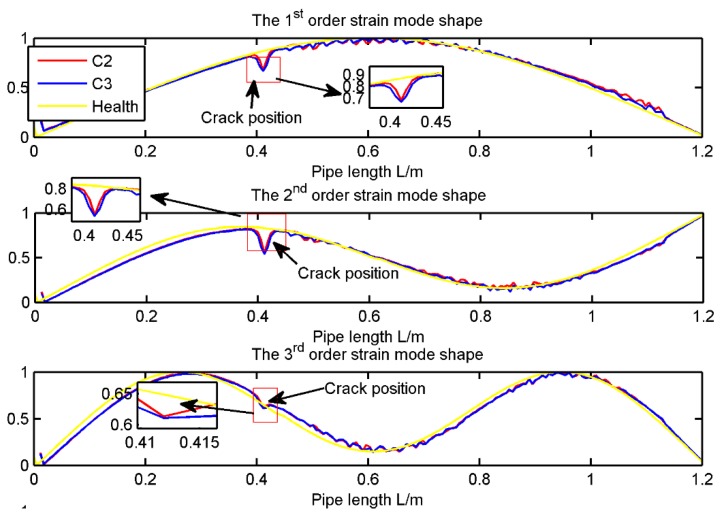
The first three strain mode shapes for all the scenarios listed in Table 4.

A three-dimensional (3D) model of structural steel pipeline with the length 1.2 m is generated in the SOLIDWORKS 2013. The physical parameters of the pipeline and the fluid are listed in Table 3. Then the 3D model is imported to ANSYS 14.0 conduct the modal analysis. The crack form is shown in Figure 5 and the detailed scenarios for the crack are listed in Table 4. Note that, the fluid in this work is the air. And the first three strain mode shape calculated from ANSYS 14.0 is shown in Figure 6.

**Figure 7:**
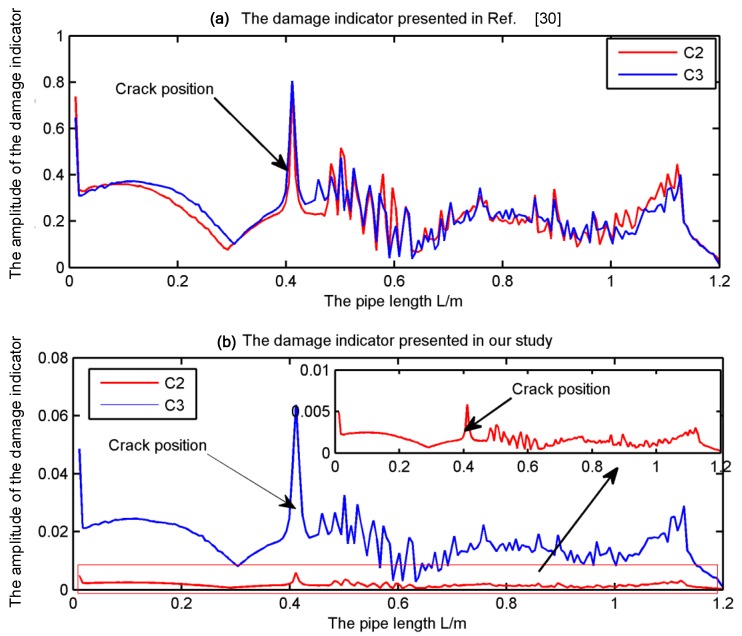
The comparison of the damage indicators presented in Reference [30] and our present study.

From Figure 6, it is clear that the strain mode shape mutation exists at the crack position. However, the scenario 2 and 3 cannot be discriminated clearly. In the following, the modal strain will be used to calculate the damage indicator presented in Reference [30] and the damage indicator in Equation (16). The specific positions to install the FBGs are shown in Section 4.3. The damage indicators presented in Reference [30] and Equation (16) for the Scenario 2 and 3 are further shown in Figure 7a,b respectively.

In Figure 7a, it is clear that the crack can be located based on the damage indicator presented in Reference [30]. However, the scenario of C2 and C3 cannot be discriminated too. However, in Figure 7b, the crack for the scenario C2 and C3 can not only be located but also can be discriminated clearly. Moreover, the damage indicator increases as the depth of the crack increases. Therefore, the presented damage indicator shows better performance than the one presented in Reference [30].

**Figure 8:**
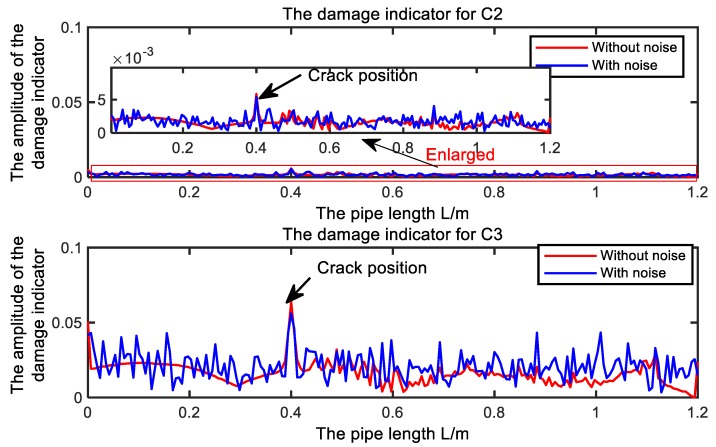
The presented damage indicators for the Scenario 2, i.e., C2 and Scenario 3, i.e., C3 with and without noise.

In order to examine the performance of the presented damage indicator shown in Equation (16) in the presence of noise in modal data, the following way to add the noise to the modal strain in Reference [50] will be employed.

(17) $$\bar{\psi_{r}^{\varepsilon }}=\psi_{r}^{\varepsilon }\cdot \left(1+\epsilon \cdot \Upsilon \right)$$

where $\bar{\psi_{r}^{\varepsilon }}$ is the contaminated modal strain, $\psi_{r}^{\varepsilon }$ is the modal strain without noise, ϵ is the random noise level and Υ is the random number with zero mean and variance of one. Similar to the work of Shi et al. [45], the modal strains are contaminated with 5% random noise in present study to consider the noise effects. Figure 8 illustrates the presented damage indicators for the C2 and C3 with and without noise.

In Figure 8, it is clear that damage indicators with noise have the similar trend as the ones without noise. The biggest damage indicators exist in the crack position too. The results show that the presented damage indicators perform well for detecting and locating the crack in the presence of 5% noise in the modal strain. The capability of the presented damage indicators will be further evaluated in the experiments.

### 4.3. Experimental Validation of the Presented Method to Detect the Crack

**Figure 9:**
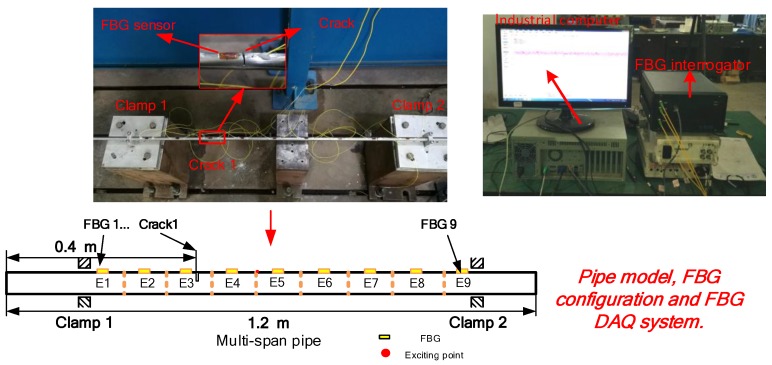
The configuration of the hydraulic pipeline test rig.

**Figure 10:**
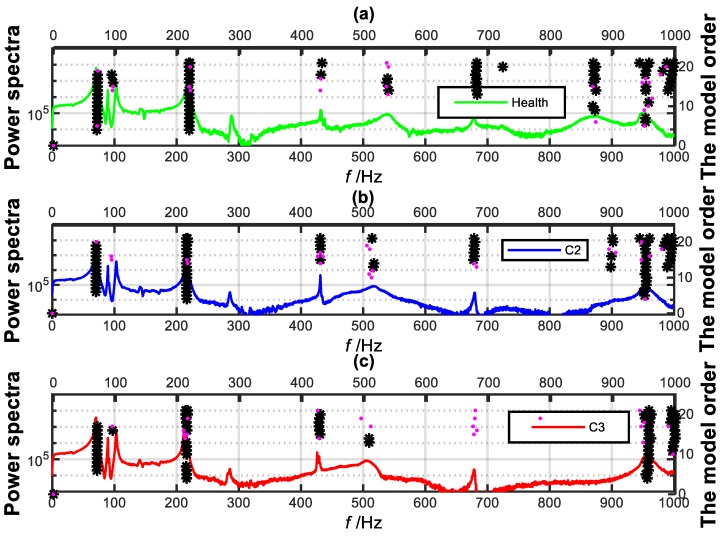
The stabilization diagram of the FBG 9. (black asterisk-frequency variation ratio [0–0.5%], Pink dot-frequency variation ratio [0.5–1%]): (**a**) The health; (**b**) the C2; (**c**) the C3.

As shown in Figure 9, one typical layout of the pipe model with single crack is constructed. Two clamps were used to support the pipe and the FBG sensors were glued on the surface of the tested pipe with 353ND glue and spaced in intervals of 100 mm along the axial direction of the pipe. As well, the FBG sensors numbered from FBG1 to FBG9. The pipe is divided into 9 elements, i.e., E1–E9 shown in Figure 9. The integrator with sampling frequency 2000 Hz is used to acquire the wavelength data of the FBG strain sensors. The physical properties of the pipe and tested scenarios are listed in Table 3. Since no ambient excitation in the laboratory, the pipe vibration is excited artificially, however, the input signal is unknown. The corresponding stabilization diagrams of the FBG9 for the tested scenarios are shown in Figure 10. The identified natural frequencies from Figure 10 are further listed in Table 5. To eliminate the effects of temperature, the detrend() function in MATLAB is employed to preprocess the FBG data.

**Figure 11:**
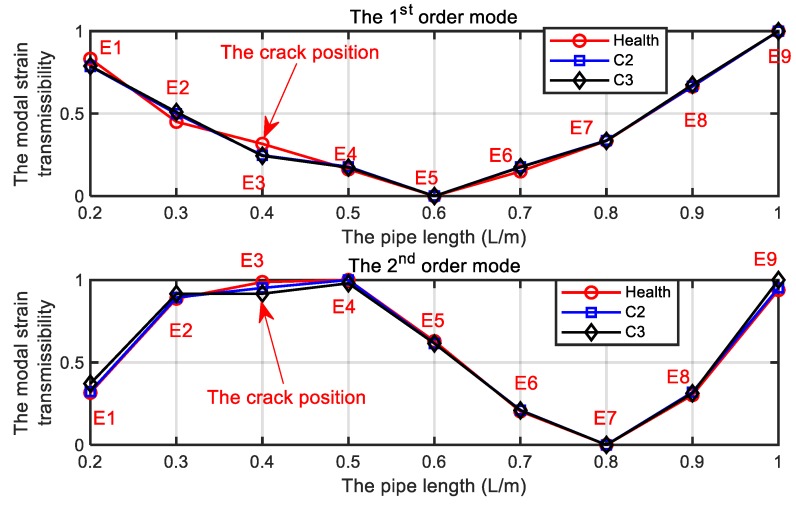
The first two modal strain transmissibility for the scenarios listed in Table 5.

**Figure 12:**
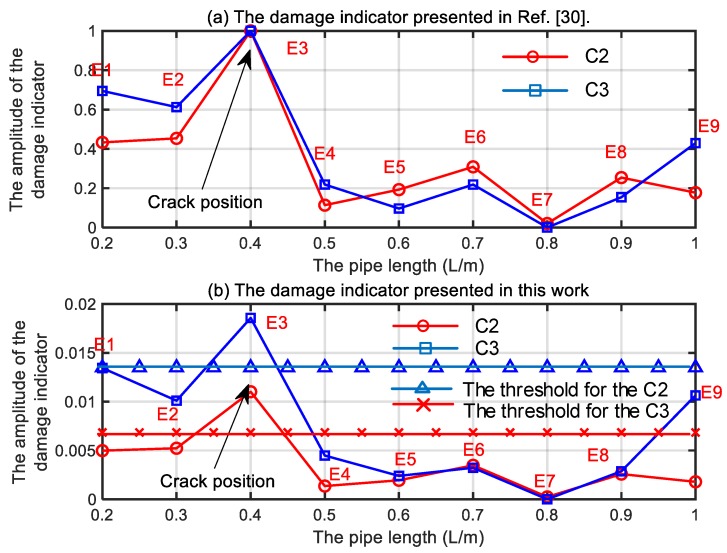
The comparison of the damage indicator from present work with damage indicator from the work of Cui et al.

From Table 5, it is clear that the natural frequencies decrease when the crack depth increases. Therefore, it is reasonable to weight the damage indicator with the change ratio of the natural frequencies shown in Equation (16). In what follows, only the first two modes are considered to calculate the damage indicator. Now we define a threshold as follows:

(18) $$\begin{array}{l} \sigma =\frac{1}{n_{o}}\overset{n_{o}}{\underset{i=1}{\sum }}\left(\frac{1}{M}\overset{M}{\underset{r=1}{\sum }}\omega_{r,\;∆}\cdot \psi_{N,\Delta r}^{\varepsilon }\left(i\right)\right)+\chi \\ \;\;\;\;\;\;\;\cdot \sqrt{\frac{1}{n_{o}-1}\cdot \overset{n_{o}}{\underset{i=1}{\sum }}\left(\frac{1}{M}\overset{M}{\underset{r=1}{\sum }}\omega_{r,\;∆}\cdot \psi_{N,\Delta r}^{\varepsilon }\left(i\right)-\frac{1}{n_{o}}\overset{n_{o}}{\underset{i=1}{\sum }}\left(\frac{1}{M}\overset{M}{\underset{r=1}{\sum }}\omega_{r,\;∆}\cdot \psi_{N,\Delta r}^{\varepsilon }\left(i\right)\right)\right)} \end{array}$$

where $n_{o}=9$ is the measurement freedom and χ is the safety coefficient. In practice, the safety coefficient should be determined by statistical methods or expert knowledge. In the present study, we just want to validate the presented method to detect and locate the cracks. Therefore, a safety coefficient χ=1 is selected to validate the presented method. The first two modal strain transmissibility for the scenarios listed in Table 4 are shown in Figure 11. The calculated damage indicators from Cui et al. [30] and from present work are further shown in Figure 12, in which the corresponding thresholds are given.

In Figure 11, it is clear that the damaged element E3 can be detected from the first two operational modal strain transmissibility. However, the C2 and C3 cannot be discernable from Figure 11 as the Figure 6 predicted. In Figure 12a, it is clear that the presented damage indicator in Ref [30] cannot discriminate the C2 and C3 effectively. In Figure 12b, the damage indicator presented in this work can discriminate the C2 and C3 effectively and the damage indicator increases as the crack depth increases. Moreover, the first two modal strain transmissibility is enough to detect and locate the crack, which can reduce the need for high-speed demodulation equipment for the FBG sensors.

## 5. Conclusions

The small and light-weight pipeline is widely used in hydraulic system for aerospace engineering. The crack is one of the most common failures in the pipelines and its incipient detection can further avoid the catastrophic damage of the piping system.

Although the modal strain based methods have been used in damage detection in some beam and plate structures, they pose some difficulties in detecting the incipient crack in small and light-weight pipeline. On one hand, the added mass effects and the wiring of traditional electrical sensors weakened the accuracy of the measurement. On the other hand, the damage indicators based on the modal strain in the literature do not consider the variation of the natural frequencies. This paper presented an enhanced damage indicator weighted by the change ratio of the natural frequencies to detect and locate the crack in the pipelines. The corresponding numerical and experimental results were used to validate the presented method. The main contributions can be drowned as follows:

(1) An FBG based OMA in our previous study was employed to obtain the modal strain of the pipeline. And the analytical model was used to validate the accuracy of the FBG based OMA.
(2) An enhanced damage indicator is presented by considering the differences of the natural frequencies and modal strain simultaneously. When compared with the damage indicator presented in [30], the damage indictor presented in this work is weighted by the change ratio of the natural frequencies, which can make the small damages discernible.
(3) The modal strain transmissibility is employed to be equivalent to modal strain in order to exclude the influence of the unknown excitation. Although the transmissibility based method has been presented to detect and locate the damages in the existing literatures [31,32,33], it has been proved that the location of the excitation and the frequency band affect the accuracy of the detection [34]. However, the modal transmissibility used in this work is independent on the excitation locations. The corresponding numerical and experimental results were conducted to validate the proposed method to detect and locate the crack.

In the future work, the metal-packaged FBG [51], the dense ultra-short FBG array with large multiplexing capacity [52,53] or the Brillouin distributed optical fiber sensors [54,55] with large scale optical fiber low-coherent interferometer [56] can be employed to enhance the capability of the presented method to locate the crack with high precision. What’s more, the presented method will be extended to detect and locate the multiple cracks in the future work.

## Figures and Tables

**Table 1:** The physical properties of the pipe model.

Parameters	Value
Lumped masses Rotational inertia Eccentricity	$m_{L}=m_{R}=0.15\;\mathrm{kg}$ $J_{L}=J_{R}=0.09\;\mathrm{kg}m^{2}$ $e_{L}=e_{R}=0\;m$
Linear springs	$K_{T,1}=K_{T,2}=10^{7}\;N/m,K_{R,1}=K_{R,2}=10^{7}\;\mathrm{Nm}/\mathrm{rad}$

**Table 2:** The comparison of the estimated natural frequencies and modal damping ratios from present OMA with the results from analytical method.

**Natural Frequencies (Hz)**				
	$f_{1}$	$f_{2}$	$f_{3}$	$f_{4}$
Proposed OMA	80.55	221	430.8	712.2
Analytical method	80.6	221.1	430.7	707.3
Relative error (%)	−1.3	−0.05	0.02	0.69
**Modal Damping Ratio**				
	$\xi_{1}$	$\xi_{2}$	$\xi_{3}$	$\xi_{4}$
Proposed OMA	−3.242 × 10^−17^	0	5.610 × 10^−14^	−0.0089
Analytical method	0	0	0	0

**Table 3:** The physical parameters of the pipeline and the fluid for simulation.

Mass density of pipe ($\mathrm{kg}/m^{3}$)	7850
Young’s elastic modulus of pipe (GPa)	200
Pipe length L (m)	1.2
Outer radius $R_{o}$ of pipe (m)	0.006
Inner radius of pipe $R_{I}\;\left(m\right)$	0.005
Pipe wall thickness δ (m)	0.001
Poisson’s ratio of the pipe	0.3
Mass density of the air $\left(\mathrm{kg}/m^{3}\right)$	1.29
The pressure of the fluid (MPa)	0
The velocity of the fluid (m/s)	0

**Table 4:** The tested scenarios.

Single Crack	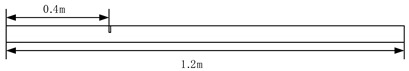	
Scenario No	Crack Depth dc (m)	Remark
1	0	Health
2	0.002	C2
3	0.003	C3

**Table 5:** The identified first four natural frequencies.

Scenario. No	Natural Frequencies (Hz)			
	$f_{1}$	$f_{2}$	$f_{3}$	$f_{4}$
1	71.23	217.8	431.6	680.4
2	70.38	215.6	431.1	678.4
3	70.35	212.4	424.9	678.2

**Figure A1:**
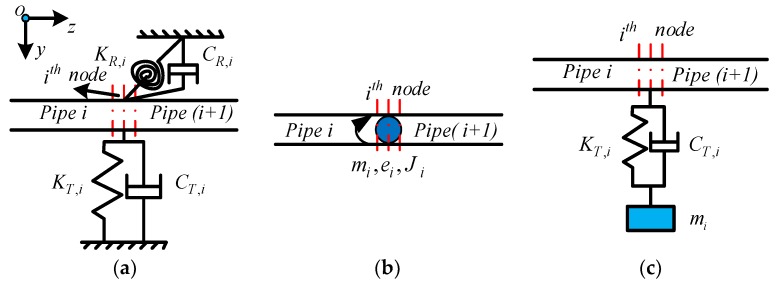
The simplified models for the accessories: (**a**) The physical model for the clamps and complex constraint; (**b**) the physical model for the valves and flanges; (**c**) the physical model for the vibration absorber.

**Figure A2:**
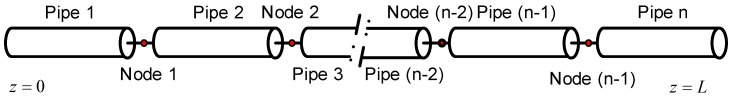
The numbering of the nodes and the segments for the cascaded pipeline.

## Appendix A

If we assume that the fluid in the pipe is steady and incompressible, the plug-flow model is used to describe the fluid and the Timoshenko beam model is employed to describe the pipe model. And then the dynamic equations of the pipe can be expressed as follows:

(A1) $$EI_{p}\cdot \left[kGA_{p}-C_{pv}\right]\cdot \frac{\partial^{4}u_{y}\left(z,t\right)}{\partial z^{4}}+Y_{1}\cdot Y_{2}\cdot \frac{\partial^{4}u_{y}\left(z,t\right)}{\partial t^{4}}-C_{V}\cdot EI_{p}\cdot \frac{\partial^{4}u_{y}\left(z,t\right)}{\partial z^{3}\partial t}\;+C_{V}\cdot Y_{2}\cdot \frac{\partial^{4}u_{y}\left(z,t\right)}{\partial z\partial t^{3}}-\left[Y_{1}\cdot EI_{p}-Y_{2}\cdot \left(C_{PV}-kGA_{p}\right)\right]\cdot \frac{\partial^{4}u_{y}\left(z,t\right)}{\partial z^{2}\partial t^{2}}\;+C_{PV}\cdot kGA_{p}\cdot \frac{\partial^{2}u_{y}\left(z,t\right)}{\partial z^{2}}+Y_{1}\cdot kGA_{p}\cdot \frac{\partial^{2}u_{y}\left(z,t\right)}{\partial t^{2}}+C_{V}\cdot kGA_{p}\cdot \frac{\partial^{2}u_{y}\left(z,t\right)}{\partial z\partial t}=0$$

(A2) $$EI_{p}\cdot \left[kGA_{p}-C_{pv}\right]\cdot \frac{\partial^{4}\theta_{x}\left(z,t\right)}{\partial z^{4}}+Y_{1}\cdot Y_{2}\cdot \frac{\partial^{4}\theta_{x}\left(z,t\right)}{\partial t^{4}}-C_{V}\cdot EI_{p}\cdot \frac{\partial^{4}\theta_{x}\left(z,t\right)}{\partial z^{3}\partial t}\;+C_{V}\cdot Y_{2}\cdot \frac{\partial^{4}\theta_{x}\left(z,t\right)}{\partial z\partial t^{3}}-\left[Y_{1}\cdot EI_{p}-Y_{2}\cdot \left(C_{PV}-kGA_{p}\right)\right]\cdot \frac{\partial^{4}\theta_{x}\left(z,t\right)}{\partial z^{2}\partial t^{2}}\;+C_{PV}\cdot kGA_{p}\cdot \frac{\partial^{2}\theta_{x}\left(z,t\right)}{\partial z^{2}}+Y_{1}\cdot kGA_{p}\cdot \frac{\partial^{2}\theta_{x}\left(z,t\right)}{\partial t^{2}}+C_{V}\cdot kGA_{p}\cdot \frac{\partial^{2}\theta_{x}\left(z,t\right)}{\partial z\partial t}=0$$

where $u_{y}\left(z,t\right)$ is the transverse deflection of the pipe at axial coordinate z and time t , E is the Young’s elastic modulus of the pipe, $I_{p}$ is the inertial section moment of the pipe, k is the shear distribution coefficient of the pipe, G is the shear elasticity modulus of pipe $A_{p}$ is the cross-sectional area of the pipe and $\theta_{x}$ is the bending slope of the pipe. And $\kappa =\frac{2\cdot \left(1+\upsilon \right)}{4+3\upsilon },\;Y_{1}=\rho_{p}\cdot A_{p}+\rho_{f}\cdot A_{f},\;Y_{2}$ = $\rho_{p}\cdot I_{p}+\rho_{f}\cdot I_{f},\;I_{p}=\frac{\pi }{4}\cdot \left({\left(R+\delta \right)}^{4}-R^{4}\right)$, $I_{f}=\frac{\pi }{4}\cdot R^{4}$, $C_{pV}=\rho_{f}A_{f}V_{f}^{2}+P_{f}A_{f}$, $C_{V}=2\cdot \rho_{f}\cdot A_{f}\cdot V_{f}$, where υ is the Poisson’s ratio of the pipe, $\rho_{p}$ is the mass density per unit volume of the pipe, $\rho_{f}$ is the mass density per unit volume of the fluid, $A_{f}$ is the cross-sectional area of the fluid, $I_{f}$ is the inertial section moment of the fluid, R is the inner radius of the pipe, δ is the wall thickness of the pipe, $V_{f}$ is the velocity of the fluid and $P_{f}$ is the pressure of the fluid.

And now we assume that the transverse displacement of the pipe $u_{y}\left(z,t\right)$ and the bending slope of the pipe $\theta_{x}\left(z,t\right)$ can be expressed as follows:

(A3) $$u_{y}\left(z,t\right)=U_{y}\left(z\right)\cdot e^{j\omega t},\;\theta_{x}\left(z,t\right)=\Theta_{x}\cdot e^{j\omega t}$$

where $U_{y}\left(z\right)$ is the amplitude of the transverse displacement, and $\Theta_{x}$ is the amplitude of the bending slope. Substitution of the Equation (A3) into Equation (A1) and Equation (A2) leads to the following equations:

(A4) $$EI_{p}\cdot \left[\kappa GA_{p}-C_{pv}\right]\cdot \frac{d^{4}U_{y}\left(z\right)}{dz^{4}}+j\omega \cdot C_{V}\cdot EI_{p}\cdot \frac{d^{3}U_{y}\left(z\right)}{dz^{3}}\;+\left\{\left[Y_{1}\cdot EI_{p}-Y_{2}\cdot \left(C_{PV}-\kappa GA_{p}\right)\right]+C_{pV}\cdot \kappa GA_{p}\right\}\cdot \frac{d^{2}U_{y}\left(z\right)}{dz^{2}}\;+j\omega \cdot C_{V}\cdot \left(\kappa GA_{p}-Y_{2}\cdot \omega^{2}\right)\cdot \frac{dU_{y}\left(z\right)}{dz}+\omega^{2}\cdot Y_{1}\cdot \left(Y_{2}\cdot \omega^{2}-kGA_{p}\right)\cdot U_{y}\left(z\right)=0$$

(A5) $$EI_{p}\cdot \left[\kappa GA_{p}-C_{pv}\right]\cdot \frac{d^{4}\Theta_{x}\left(z\right)}{dz^{4}}+j\omega \cdot C_{V}\cdot EI_{p}\cdot \frac{d^{3}\Theta_{x}\left(z\right)}{dz^{3}}\;+\left\{\left[Y_{1}\cdot EI_{p}-Y_{2}\cdot \left(C_{PV}-\kappa GA_{p}\right)\right]+C_{pV}\cdot \kappa GA_{p}\right\}\cdot \frac{d^{2}\Theta_{x}\left(z\right)}{dz^{2}}\;+j\omega \cdot C_{V}\cdot \left(\kappa GA_{p}-Y_{2}\cdot \omega^{2}\right)\cdot \frac{d\Theta_{x}\left(z\right)}{dz}+\omega^{2}\cdot Y_{1}\cdot \left(Y_{2}\cdot \omega^{2}-\kappa GA_{p}\right)\cdot \Theta_{x}\left(z\right)=0$$

The solution of the Equations (A4) and (A5) can be obtained as follows:

(A6) $$U_{y}\left(z\right)=A_{1}\cdot cosh\left(\sigma \cdot z\right)+A_{2}\cdot sinh\left(\sigma \cdot z\right)+A_{3}\cdot cos\left(\gamma \cdot z\right)+A_{4}\cdot sin\left(\gamma \cdot z\right)$$

(A7) $$\Theta_{x}\left(z\right)=\bar{A_{1}}\cdot cosh\left(\sigma \cdot z\right)+\bar{A_{2}}\cdot sinh\left(\sigma \cdot z\right)+\bar{A_{3}}\cdot cos\left(\gamma \cdot z\right)+\bar{A_{4}}\cdot sin\left(\gamma \cdot z\right)$$

where $\sigma =\sqrt{\frac{1}{2}\left(-\alpha +\sqrt{4\beta^{4}+\alpha^{2}}\right)}$, $\gamma =\sqrt{\frac{1}{2}\left(\alpha +\sqrt{4\beta^{4}+\alpha^{2}}\right)}$, and $\alpha =\frac{\left[Y_{1}\cdot EI_{p}+Y_{2}\cdot \left(\kappa GA_{p}-C_{pV}\right)\right]+C_{PV}\cdot kGA_{p}}{EI_{p}\cdot \left(kGA_{p}-C_{pV}\right)}$, $\beta^{4}=\frac{Y_{1}\cdot \omega^{2}\cdot \left(\kappa GA_{p}-Y_{2}\cdot \omega^{2}\right)}{EI_{p}\cdot \left(kGA_{p}-C_{pV}\right)}$.

If we define $A={\left[\begin{array}{cc} A_{1} & \begin{array}{ccc} A_{2} & A_{3} & A_{4} \end{array} \end{array}\right]}^{T}$ and $\bar{A}={\left[\begin{array}{cc} \bar{A_{1}} & \begin{array}{ccc} \bar{A_{2}} & \bar{A_{3}} & \bar{A_{4}} \end{array} \end{array}\right]}^{T}$, where $\bar{A_{1}}=q\cdot A_{2},\;\bar{\;A_{2}}=q\cdot A_{1},\;\;\bar{A_{3}}=\bar{q}\cdot A_{4},\;\bar{A_{4}}=-\bar{q}\cdot A_{3}$ then the Equation (12) and Equation (13) can be expressed as follows:

(A8) $$U_{y}\left(z\right)=U_{y}\left(z\right)\cdot A,\;\Theta_{x}\left(z\right)=\Theta_{x}\left(z\right)\cdot A$$

where

(A9) $$U_{y}\left(z\right)=\left[\begin{array}{cc} cosh\left(\sigma \cdot z\right) & \begin{array}{ccc} sinh\left(\sigma \cdot z\right) & cos\left(\gamma \cdot z\right) & sin\left(\gamma \cdot z\right) \end{array} \end{array}\right]$$

(A10) $$\Theta_{x}\left(z\right)=\left[\begin{array}{cc} q\cdot sinh\left(\sigma \cdot z\right) & q\cdot \begin{array}{ccc} cosh\left(\sigma \cdot z\right) & -\bar{q}\cdot sin\left(\gamma \cdot z\right) & \bar{q}\cdot cos\left(\gamma \cdot z\right) \end{array} \end{array}\right]$$

As we all know that the accessories, i.e., valves, clamps, vibration absorbers, flanges and complex constraints are widely used in the occasions of piping system. The simplified models for the accessories are shown in Figure A1. And we assume that the discontinuities induced by the accessories are located at the $i^{th}$ node. The model in Figure A1a is used to model the clamps and the complex constraints, the Figure A1b represents the simplified model of the valves, pipe connectors and flanges, and vibration absorber is modelled in Figure A1c.

If we introduce the equivalent translational stiffness $K_{eT,i}\;$, equivalent rotational stiffness $K_{eR,i}$, equivalent translational damping coefficient $C_{eT,i}$ and equivalent rotational damping coefficient $C_{eR,\;i}$ and $\Gamma_{i}$ for the models in Figure A1, then the compatibility and the equilibrium conditions at the $i^{th}$ node can be formulated in a general way as follows:

(A11) $$U_{y,i}\left(z_{i}\right)=U_{y,i+1}\left(z_{i}\right)$$

(A12) $$\Theta_{x,i}\left(z_{i}\right)=\Theta_{x,i+1}\left(z_{i}\right)$$

(A13) $$\begin{array}{ll} E_{i}I_{p,i}\cdot \frac{d\Theta_{x,i}\left(z_{i}\right)}{dz} & +K_{eR,i}\cdot \Theta_{x,i}\left(z_{i}\right)+j\omega \cdot C_{eR,i}\cdot \Theta_{x,i}\left(z_{i}\right)+\Gamma_{i}\cdot U_{y,i}\left(z_{i}\right)\\ & =E_{i+1}I_{p,i+1}\cdot \frac{d\Theta_{x,i+1}\left(z_{i}\right)}{dz} \end{array}$$

(A14) $$\begin{array}{c} \kappa_{i}G_{i}A_{p,i}\cdot \left(\frac{dU_{y,i}\left(z_{i}\right)}{dz}-\Theta_{x,i}\left(z_{i}\right)\right)+K_{eT,i}\cdot U_{y,i}\left(z_{i}\right)+j\omega \cdot C_{eT,i}\cdot U_{y,i}\left(z_{i}\right)+\Gamma_{i}\cdot \Theta_{x,i}\left(z_{i}\right)\\ =\kappa_{i+1}\cdot G_{i+1}\cdot A_{p,i+1}\cdot \left(\frac{dU_{y,i+1}\left(z_{i}\right)}{dz}-\Theta_{x,i+1}\left(z_{i}\right)\right) \end{array}$$

Specifically, for the model in Figure A1a $K_{eT,i}=K_{T,i},\;C_{eT,i}=C_{T,i},\;K_{eR,i}=K_{R,i},\;C_{eR,i}=C_{R,i}$ and $\Gamma_{i}=0,$ in Figure A1b $K_{eT,i}=-m_{i}\omega^{2},\;K_{eR,i}=-{\left(J_{i}+m_{i}\cdot e_{i}^{2}\right)}^{2},\;C_{eR,i}=C_{eT,i}=0,\;\Gamma_{i}=m_{i}\cdot e_{i}\cdot \omega^{2}$, in $K_{eT,i}=-\frac{K_{T,i}\cdot m_{i}\omega^{2}\cdot \left(K_{T,i}-m_{i}\omega^{2}\right)+\omega^{2}\cdot C_{T,i}^{2}}{\left(K_{T,i}-m_{i}\cdot \omega^{2}\right)+\omega^{2}\cdot C_{T,i}^{2}}$, $C_{eT,i}=\frac{m_{i}\omega^{2}\cdot C_{T,i}^{2}}{{\left(K_{T,i}-m_{i}\omega^{2}\right)}^{2}+\omega^{2}\cdot C_{T,i}^{2}}$, $K_{eR,i}=C_{eR,i}=\Gamma_{i}=0$, where $K_{T,i}$ is the stiffness of the translational spring, $C_{T,i}$ is the damping of the translational damper, $K_{R,i}$ is the rotational stiffness of the rotational spring, $C_{R,i}$ is the rotational damping of the rotational damper, $m_{i}$ is the added mass, $e_{i}$ is the eccentricity of the added mass $m_{i}$, and $J_{i}$ is the rotary inertia of the added mass $m_{i}$. Substitutions of Equation (A8) into Equations (A11)–(A14) lead to the following equation:

(A15) $$T_{\left(i\right)}\cdot A_{i}=T_{\left(i+1\right)}\cdot A_{i+1}$$

where

(A16) $$A_{i}={\left[\begin{array}{cc} A_{1,i} & \begin{array}{ccc} A_{2,i} & A_{3,i} & A_{4,i} \end{array} \end{array}\right]}^{T},\;A_{i+1}={\left[\begin{array}{cc} A_{1,i+1} & \begin{array}{ccc} A_{2,i+1} & A_{3,i+1} & A_{4,i+1} \end{array} \end{array}\right]}^{T}$$

(A17) $$T_{\left(i+1\right)}=\left[\begin{array}{cccc} 1 & 0 & 1 & 0\\ 0 & q_{i+1} & 0 & {\bar{q}}_{i+1}\\ \zeta_{1} & 0 & -\zeta_{2} & 0\\ 0 & \zeta_{3} & 0 & \zeta_{4} \end{array}\right],\;T_{\left(i\right)}=\left[\begin{array}{c} \begin{array}{cc} T_{11}^{\left(i\right)} & \begin{array}{ccc} T_{12}^{\left(i\right)} & T_{13}^{\left(i\right)} & T_{14}^{\left(i\right)} \end{array} \end{array}\\ \begin{array}{c} \begin{array}{cc} T_{21}^{\left(i\right)} & \begin{array}{ccc} T_{22}^{\left(i\right)} & T_{23}^{\left(i\right)} & T_{24}^{\left(i\right)} \end{array} \end{array}\\ \begin{array}{cc} T_{31}^{\left(i\right)} & \begin{array}{ccc} T_{32}^{\left(i\right)} & T_{33}^{\left(i\right)} & T_{34}^{\left(i\right)} \end{array} \end{array}\\ \begin{array}{cc} T_{41}^{\left(i\right)} & \begin{array}{ccc} T_{42}^{\left(i\right)} & T_{43}^{\left(i\right)} & T_{44}^{\left(i\right)} \end{array} \end{array} \end{array} \end{array}\right]$$

(A18) $$\zeta_{1}=E_{i+1}\cdot I_{p,i+1}\cdot q_{i+1}\cdot \sigma_{i+1},\;\zeta_{2}=E_{i+1}\cdot I_{p,i+1}\cdot {\bar{q}}_{i+1}\cdot \gamma_{i+1}\;\zeta_{3}=\kappa_{i+1}\cdot G_{i+1}A_{p,i+1}\cdot \left(\sigma_{i+1}-q_{i+1}\right),\;\zeta_{4}=\kappa_{i+1}\cdot G_{i+1}\cdot A_{p,i+1}\cdot \left(\gamma_{i+1}-{\bar{q}}_{i+1}\right)$$

where

$T_{11}^{\left(i\right)}=cosh\left(\sigma_{i}\cdot l_{i}\right),\;T_{12}^{\left(i\right)}=sinh\left(\sigma_{i}\cdot l_{i}\right),\;T_{13}^{\left(i\right)}=cos\left(\gamma_{i}\cdot l_{i}\right),\;T_{14}^{\left(i\right)}=sin\left(\gamma_{i}\cdot l_{i}\right)$
$T_{21}^{\left(i\right)}=q_{i}\cdot sinh\left(\sigma_{i}\cdot l_{i}\right),\;T_{22}^{\left(i\right)}=q_{i}\cdot cosh\left(\sigma_{i}\cdot l_{i}\right),\;T_{23}^{\left(i\right)}=-{\bar{q}}_{i}\cdot sin\left(\gamma_{i}\cdot l_{i}\right),\;T_{24}^{\left(i\right)}={\bar{q}}_{i}\cdot cos\left(\gamma_{i}\cdot l_{i}\right)$
$T_{31}^{\left(i\right)}=\left(E_{i}\cdot I_{p,i}\cdot q_{i}+\Gamma_{i}\right)\cdot cosh\left(\sigma_{i}\cdot l_{i}\right)+\left(K_{eR,i}+j\omega \cdot C_{eR,i}\right)\cdot q_{i}\cdot sinh\left(\sigma_{i}\cdot l_{i}\right)$
$T_{32}^{\left(i\right)}=\left(E_{i}\cdot I_{p,i}\cdot q_{i}+\Gamma_{i}\right)\cdot sinh\left(\sigma_{i}\cdot l_{i}\right)+\left(K_{eR,i}+j\omega \cdot C_{eR,i}\right)\cdot q_{i}\cdot cosh\left(\sigma_{i}\cdot l_{i}\right)$
$T_{33}^{\left(i\right)}=\left(-E_{i}\cdot I_{p,i}\cdot {\bar{q}}_{i}+\Gamma_{i}\right)\cdot cos\left(\gamma_{i}\cdot l_{i}\right)-\left(K_{eR,i}+j\omega \cdot C_{eR,i}\right)\cdot {\bar{q}}_{i}\cdot sinh\left(\gamma_{i}\cdot l_{i}\right)$
$T_{34}^{\left(i\right)}=\left(-E_{i}\cdot I_{p,i}\cdot {\bar{q}}_{i}+\Gamma_{i}\right)\cdot sin\left(\gamma_{i}\cdot l_{i}\right)+\left(K_{eR,i}+j\omega \cdot C_{eR,i}\right)\cdot {\bar{q}}_{i}\cdot cos\left(\gamma_{i}\cdot l_{i}\right)$
$T_{41}^{\left(i\right)}=\left(\kappa_{i}G_{i}A_{p,i}\left(\sigma_{i}-q_{i}\right)+\Gamma_{i}\cdot q_{i}\right)sinh\left(\sigma_{i}\cdot l_{i}\right)+\left(K_{eT,i}+j\omega \cdot C_{eT,i}\right)\cdot cosh\left(\sigma_{i}\cdot l_{i}\right)$
$T_{42}^{\left(i\right)}=\left(\kappa_{i}G_{i}A_{p,i}\left(\sigma_{i}-q_{i}\right)+\Gamma_{i}\cdot q_{i}\right)cosh\left(\sigma_{i}\cdot l_{i}\right)+\left(K_{eT,i}+j\omega \cdot C_{eT,i}\right)\cdot sinh\left(\sigma_{i}\cdot l_{i}\right)$
$T_{43}^{\left(i\right)}=\left(-\kappa_{i}G_{i}A_{p,i}\left(\gamma_{i}-{\bar{q}}_{i}\right)-\Gamma_{i}\cdot {\bar{q}}_{i}\right)sin\left(\gamma_{i}\cdot l_{i}\right)+\left(K_{eT,i}+j\omega \cdot C_{eT,i}\right)\cdot cos\left(\gamma_{i}\cdot l_{i}\right)$
$T_{44}^{\left(i\right)}=\left(\kappa_{i}G_{i}A_{p,i}\left(\gamma_{i}-{\bar{q}}_{i}\right)+\Gamma_{i}\cdot {\bar{q}}_{i}\right)cos\left(\gamma_{i}\cdot l_{i}\right)+\left(K_{eT,i}+j\omega \cdot C_{eT,i}\right)\cdot sin\left(\gamma_{i}\cdot l_{i}\right)$

Based on the Equation (A15), the following equation can be obtained.

(A19) $$A_{i+1}={\left[T_{\left(i+1\right)}\right]}^{-1}\cdot T_{\left(i\right)}\cdot A_{i}={\bar{T}}_{e}^{i}\cdot A_{i}$$

We assume that the mentioned accessories in Figure A1 exist in the ends of the pipe, the boundary conditions can be formulated in a general way as follows:

(A20) $$R_{2\times 4}^{\left(L\right)}\cdot A_{4\times 1}^{\left(L\right)}=0$$

(A21) $$R_{2\times 4}^{\left(R\right)}\cdot A_{4\times 1}^{\left(R\right)}=0$$

where $R_{2\times 4}^{\left(L\right)}=\left[\begin{array}{cc} \begin{array}{c} R_{11}^{\left(L\right)}\\ R_{21}^{\left(L\right)} \end{array} & \begin{array}{ccc} \begin{array}{c} R_{12}^{\left(L\right)}\\ R_{22}^{\left(L\right)} \end{array} & \begin{array}{c} R_{13}^{\left(L\right)}\\ R_{23}^{\left(L\right)} \end{array} & \begin{array}{c} R_{14}^{\left(L\right)}\\ R_{24}^{\left(L\right)} \end{array} \end{array} \end{array}\right]$ and $R_{2\times 4}^{\left(Rs\right)}=\left[\begin{array}{cc} \begin{array}{c} R_{11}^{\left(R\right)}\\ R_{21}^{\left(R\right)} \end{array} & \begin{array}{ccc} \begin{array}{c} R_{12}^{\left(R\right)}\\ R_{22}^{\left(R\right)} \end{array} & \begin{array}{c} R_{13}^{\left(R\right)}\\ R_{23}^{\left(R\right)} \end{array} & \begin{array}{c} R_{14}^{\left(R\right)}\\ R_{24}^{\left(R\right)} \end{array} \end{array} \end{array}\right]$ can be expressed as follows:

$R_{11}^{\left(L\right)}=E_{1}\cdot I_{p,1}\cdot q_{1}\cdot \sigma_{1}-\Gamma_{0}\left(0\right),\;R_{12}^{\left(Ls\right)}=-\left(K_{eR,0}+j\omega \cdot C_{R,0}\right)\cdot q_{1}$
$R_{13}^{\left(L\right)}=E_{1}\cdot I_{p,1}\cdot {\bar{q}}_{1}\cdot \gamma_{1}+\Gamma_{0}\left(0\right),\;R_{14}^{\left(Ls\right)}=-\left(K_{eR,0}+j\omega \cdot C_{R,0}\right)\cdot {\bar{q}}_{1}$
$R_{21}^{\left(L\right)}=-\left(K_{eT,0}+j\omega \cdot C_{T,0}\right),\;R_{22}^{\left(Ls\right)}=k_{1}G_{1}A_{p,1}\cdot \left(\sigma_{1}-q_{1}\right)-\Gamma_{0}\cdot q_{1}$
$R_{23}^{\left(L\right)}=-\left(K_{eT,0}+j\omega \cdot C_{T,0}\right),\;R_{24}^{\left(Ls\right)}=k_{1}G_{1}A_{p,1}\cdot \left(\gamma_{1}-{\bar{q}}_{1}\right)-\Gamma_{0}\cdot {\bar{q}}_{1}$
$R_{11}^{\left(R\right)}=\left(E_{n}\cdot I_{p,n}\cdot q_{n}\cdot \sigma_{n}-\Gamma_{n}\left(l_{n}\right)\right)\cdot cosh\left(\sigma_{n}\cdot l_{n}\right)+\left(K_{eR,n}+j\omega \cdot C_{R,n}\right)\cdot q_{n}\cdot sinh\left(\sigma_{n}\cdot l_{n}\right)$
$R_{12}^{\left(R\right)}=\left(E_{n}\cdot I_{p,n}\cdot q_{n}\cdot \sigma_{n}+\Gamma_{n}\left(l_{n}\right)\right)\cdot sinh\left(\sigma_{n}\cdot l_{n}\right)+\left(K_{eR,n}+j\omega \cdot C_{R,n}\right)\cdot q_{n}\cdot cosh\left(\sigma_{n}\cdot l_{n}\right)$
$R_{13}^{\left(R\right)}=\left(\Gamma_{n}\left(l_{n}\right)-E_{n}\cdot I_{p,n}\cdot {\bar{q}}_{n}\cdot \gamma_{n}\right)cos\left(\gamma_{n}\cdot l_{n}\right)-\left(K_{eR,n}+j\omega \cdot C_{eR,n}\right)\cdot {\bar{q}}_{n}\cdot sin\left(\gamma_{n}\cdot l_{n}\right)$
$R_{14}^{\left(R\right)}=\left(\Gamma_{n}\left(l_{n}\right)-E_{n}\cdot I_{p,n}\cdot {\bar{q}}_{n}\cdot \gamma_{n}\right)sin\left(\gamma_{n}\cdot l_{n}\right)+\left(K_{eR,n}+j\omega \cdot C_{eR,n}\right)\cdot {\bar{q}}_{n}\cdot cos\left(\gamma_{n}\cdot l_{n}\right)$
$R_{21}^{\left(R\right)}=\left(k_{n}\cdot G_{n}\cdot A_{p,n}\cdot \left(\sigma_{n}-q_{n}\right)+\Gamma_{n}\left(l_{n}\right)\cdot q_{n}\right)\cdot sinh\left(\sigma_{n}\cdot l_{n}\right)+\left(K_{eT,n}+j\omega \cdot C_{eT,n}\right)\cdot cosh\left(\sigma_{n}\cdot l_{n}\right)$
$R_{22}^{\left(R\right)}=\left(k_{n}\cdot G_{n}\cdot A_{p,n}\cdot \left(\sigma_{n}-q_{n}\right)+\Gamma_{n}\left(l_{n}\right)\cdot q_{n}\right)\cdot cosh\left(\sigma_{n}\cdot l_{n}\right)+\left(K_{eT,n}+j\omega \cdot C_{eT,n}\right)\cdot sinh\left(\sigma_{n}\cdot l_{n}\right)$
$R_{23}^{\left(R\right)}=-\left(k_{n}\cdot G_{n}\cdot A_{p,n}\cdot \left(\gamma_{n}-{\bar{q}}_{n}\right)+\Gamma_{n}\left(l_{n}\right)\cdot {\bar{q}}_{n}\right)\cdot sin\left(\gamma_{n}\cdot l_{n}\right)+\left(K_{eT,n}+j\omega \cdot C_{eT,n}\right)\cdot cos\left(\gamma_{n}\cdot l_{n}\right)$
$R_{24}^{\left(R\right)}=\left(k_{n}\cdot G_{n}\cdot A_{p,n}\cdot \left(\gamma_{n}-{\bar{q}}_{n}\right)+\Gamma_{n}\left(l_{n}\right)\cdot {\bar{q}}_{n}\right)\cdot cos\left(\gamma_{n}\cdot l_{n}\right)+\left(K_{eT,n}+j\omega \cdot C_{eT,n}\right)\cdot sin\left(\gamma_{n}\cdot l_{n}\right)$

The numbering of the nodes and the segments for the pipeline are shown in Figure A2.

Based on the TMM, the following equation can be obtained.

(A22) $$A_{4\times 1}^{\left(R\right)}={\bar{T}}_{e}^{\left(n-1\right)}\cdot {\bar{T}}_{e}^{\left(n-2\right)}\cdots {\bar{T}}_{e}^{\left(2\right)}\cdot {\bar{T}}_{e}^{\left(1\right)}\cdot A_{4\times 1}^{\left(L\right)}=U_{4\times 4}\cdot A_{4\times 1}^{\left(L\right)}$$

Substitution of Equation (A22) into Equation (A21) yields the following equation.

(A23) $$R_{2\times 4}^{\left(R\right)}\cdot U_{4\times 4}\cdot A_{4\times 1}^{\left(L\right)}=G_{2\times 4}\cdot A_{4\times 1}^{\left(L\right)}=0$$

Combing the Equation (A20) and Equation (A23) one can obtain the following equation:

(A24) $$\left[\begin{array}{c} G_{2\times 4}\\ R_{2\times 4}^{\left(LH\right)} \end{array}\right]\cdot A_{4\times 1}^{\left(L\right)}=0$$

The non-trial solution of Equation (A24) requires that the following coefficient determinant should be zero.

(A25) $$det\left(\left[\begin{array}{c} G_{2\times 4}\\ R_{2\times 4}^{\left(LH\right)} \end{array}\right]\right)=0$$

From Equation (A25) the natural frequencies can be obtained. Once the natural frequencies are obtained, the mode shapes can be obtained from Equation (A24). In order to calculate the vibration response of the pipe under the random load, in what follows, the pseudo excitation method (PEM) will be employed [49]. It is well known that the power spectral density (PSD) of the response for a time-invariant system can be expressed as follows:

(A26) $$S_{YY}\left(\omega \right)=H_{Y}^{*}\left(\omega \right)\cdot H_{Y}\left(\omega \right)\cdot S_{XX}\left(\omega \right)={\left|H_{Y}\left(\omega \right)\right|}^{2}S_{XX}\left(\omega \right)$$

The response, i.e., y(t) of the linear system under a harmonic excitation $x\left(t\right)=F_{x}\cdot e^{j\omega t}$ can be expressed as follows:

(A27) $$y\left(t\right)=H\left(\omega \right)\cdot F_{x}\cdot e^{j\omega t}$$

where $F_{x}$ is the amplitude for the input. If the harmonic excitation is replaced by a pseudo excitation as follows:

(A28) $$\tilde{x}\left(t\right)=\sqrt{S_{XX}\left(\omega \right)}\cdot e^{j\omega t}$$

Then the pseudo response can be obtained as follows:

(A29) $$\tilde{y}\left(t\right)=H\left(\omega \right)\cdot \sqrt{S_{XX}\left(\omega \right)}\cdot e^{j\omega t}$$

From Equation (A29) the following equation can be obtained as follows:

(A30) $$\tilde{y}\left(t\right)\cdot {\left(\tilde{y}\left(t\right)\right)}^{*}=S_{XX}\left(\omega \right)\cdot {\left|H\left(\omega \right)\right|}^{2}$$

where the superscript ∗ is the complex conjugate operator. Combining the Equation (A26) and Equation (A30), one can obtain the PSD of the response as follows:

(A31) $$S_{YY}\left(\omega \right)=\tilde{y}\left(t\right)\cdot {\left(\tilde{y}\left(t\right)\right)}^{*}$$

And now we assume a pseudo excitation numbered by u (1≤u≤n) applied on the pipe, the compatibility and equilibrium conditions at the position of the excitation can be obtained as follows:

(A32) $$A_{u+1}={\bar{T}}_{e}^{\left(u\right)}\cdot A_{u}+{\left[T_{u+1}\right]}^{-1}\cdot F_{u}$$

where $F_{u}={\left[\begin{array}{ccc} 0 & 0 & \begin{array}{cc} \sqrt{S_{M_{X},M_{X},u}} & \sqrt{S_{Q_{X},Q_{X},u}} \end{array} \end{array}\;\right]}^{T}$, the elements $\sqrt{S_{M_{X},M_{X},u}}$ and $\sqrt{S_{Q_{X},Q_{X},u}}$ are the amplitude of the pseudo excitation $\sqrt{S_{M_{X},M_{X},u}}\cdot e^{j\omega t}$ and $\sqrt{S_{Q_{X},Q_{X},u}}\cdot e^{j\omega t}$ respectively. $S_{M_{X},M_{x},u}$ represents the self-PSD of the bending moment $M_{x,u}$ and $S_{Q_{X},Q_{X},u}$ is the self-PSD of the shear force $Q_{x,u}$. Now the Equation (A22) should be changed as follows:

(A33) $$A_{4\times 1}^{\left(R\right)}=\underset{4\times 4}{\underbrace{\left(\overset{\left(n-1\right)}{\underset{m=1}{\prod }}{\bar{T}}_{e}^{\left(n-m\right)}\right)}}\cdot A_{4\times 1}^{\left(L\right)}+\underset{4\times 4}{\underbrace{\left(\overset{\left(n-1-u\right)}{\underset{m=1}{\prod }}{\bar{T}}_{e}^{\left(n-m\right)}\right)}}\cdot {\underset{4\times 4}{\underbrace{\left[T_{u+1}\right]}}}^{-1}\cdot F_{u}=U_{4\times 4}\cdot A_{4\times 1}^{\left(L\right)}+{∆}_{u}\cdot F_{u}$$

Combining the Equation (A20), Equation (A23) and Equation (A33), one can obtain the following equation:

(A34) $$A_{4\times 1}^{\left(L\right)}=\underset{4\times 4}{\underbrace{{\left[\begin{array}{c} R_{2\times 4}^{\left(L\right)}\\ G_{2\times 4} \end{array}\right]}^{-1}}\cdot }\underset{4\times 1}{\underbrace{\left[\begin{array}{c} 0_{2\times 1}\\ -R_{2\times 4}^{\left(RH\right)}\cdot {∆}_{u}\cdot F_{u} \end{array}\right]}}$$

Combing the Equation (A8) and Equation (A34), the pseudo response of the transverse displacement and bending slope can be obtained as follows:

(A35) $$\left[\begin{array}{c} U_{y,1}\left(z\right)\\ \Theta_{x,1}\left(z\right) \end{array}\right]=\left[\begin{array}{c} \Phi_{1}\left(z\right)\\ \Psi_{1\left(z\right)} \end{array}\right]\underset{4\times 4}{\underbrace{{\left[\begin{array}{c} R_{2\times 4}^{\left(LH\right)}\\ G_{2\times 4} \end{array}\right]}^{-1}}\cdot }\underset{4\times 1}{\underbrace{\left[\begin{array}{c} 0_{2\times 1}\\ -R_{2\times 4}^{\left(RH\right)}\cdot {∆}_{u}\cdot F_{u} \end{array}\right]}}$$

Based on the TMM, the pseudo response for the $i^{th}$ (2≪i≪n) pipe segment at the position of z can be obtained as follows:

(A36) $$\begin{array}{l} \left[\begin{array}{c} U_{y,i}\left(\omega ,\;z\right)\\ \Theta_{x,i}\left(\omega ,z\right) \end{array}\right]\\ =\{\begin{array}{c} \underset{2\times 4}{\underbrace{\left[\begin{array}{c} \Phi_{1}\left(z\right)\\ \Psi_{1\left(z\right)} \end{array}\right]}}\cdot \underset{4\times 4}{\underbrace{\left(\overset{i-1}{\underset{m=1}{\prod }}{\bar{T}}_{e}^{\left(i-m\right)}\right)}}\cdot A_{4\times 1}^{\left(L\right)},\;\left(2\leq i\ll u\right)\\ \underset{2\times 4}{\underbrace{\left[\begin{array}{c} \Phi_{1}\left(z\right)\\ \Psi_{1\left(z\right)} \end{array}\right]}}\cdot \left\{\underset{4\times 4}{\underbrace{\left(\overset{i-1}{\underset{m=1}{\prod }}{\bar{T}}_{e}^{\left(i-m\right)}\right)}}\cdot A_{4\times 1}^{\left(L\right)}+\underset{4\times 4}{\underbrace{\left(\overset{\left(i-1-u\right)}{\underset{m=1}{\prod }}{\bar{T}}_{e}^{\left(i-m\right)}\right)}}\cdot {\underset{4\times 4}{\underbrace{\left[T_{u+1}\right]}}}^{-1}\cdot \underset{4\times 1}{\underbrace{F_{u}}}\right\},\;\left(u+1\leq i\leq n\right) \end{array} \end{array}$$

Based on Equation (A31) and Equation (A36), the PSD for the output $u_{y,i}\left(z,t\right)$ and $\Theta_{x,i}\left(z,t\right)$ can be obtained as follows:

(A37) $$S_{UY,\;\;UY,i}\left(z,\omega \right)=U_{y,i}\left(\omega ,z\right)\cdot {\left(U_{y,i}\left(\omega ,z\right)\right)}^{*}\;S_{\Theta x,\;\;\Theta x,i}\left(z,\omega \right)=\Theta_{x,i}\left(\omega ,z\right)\cdot {\left(\Theta_{x,i}\left(\omega ,z\right)\right)}^{*}$$

The axial strain of the pipe surface can be expressed as follows:

(A38) $$\varepsilon \left(z,t\right)=\left(R+\frac{\delta }{2}\right)\cdot \frac{d\theta_{x}\left(z,t\right)}{dz}$$

Substitution of Equation (A3) into Equation (A38) yields the following equation:

(A39) $$\psi_{r,i}^{\varepsilon }\left(z,\omega \right)=\left(R+\frac{\delta }{2}\right)\cdot \frac{d\Theta_{x,i}\left(z,\omega \right)}{dz}$$

The PSD for the strain can be obtained based on Equation (A38), Equation (A10), Equation (A34), Equation (A31) and the TMM.
